# Effect of marital status on the survival outcomes of cervical cancer: a retrospective cohort study based on SEER database

**DOI:** 10.1186/s12905-024-02907-5

**Published:** 2024-01-28

**Authors:** Qing Chen, Jinyan Zhao, Xiang Xue, Xiuying Xie

**Affiliations:** Department of Obstetrics and Gynecology, The Second Affiliated Hospital of Xi’an Jiaotong University, Xi’an, 710004 P.R. China

**Keywords:** Cervical cancer, Cox regression model, Competing risk model, Marital status, SEER

## Abstract

**Background:**

Cervical cancer is the fourth most common malignant tumor troubling women worldwide. Whether marital status affects the prognosis of cervical cancer is still unclear. Here, we investigate the prognostic value of marital status in patients with cervical cancer based on the seer database.

**Material/Methods:**

The demographic and clinical data of patients with cervical cancer were extracted from the Surveillance, Epidemiology, and End Results (SEER) database from 1975 to 2017. Patients were divided into two groups (married and unmarried) according to marital status, and then the clinical characteristics of each group were compared using the chi-square test. Propensity score matching (PSM) was used to reduce differences in baseline characteristics. The overall survival (OS) and cervical cancer-specific survival (CCSS) were assessed by the Kaplan-Meier method, univariate and multivariate Cox regression models, and stratified analysis. Moreover, univariate and multivariate competing risk regression models were performed to calculate hazard ratios (HR) of death risk.

**Results:**

A total of 21,148 patients were included in this study, including 10,603 married patients and 10,545 unmarried patients. Married patients had better OS(*P* < 0.05) and CCSS (*P* < 0.05) compared to unmarried patients, and marital status was an independent prognostic factor for both OS (HR: 0.830, 95% CI: 0.798–0.862) and CCSS (HR: 0.892, 95% CI: 0.850–0.937). Moreover, after eliminating the competing risk, married patients (CCSD: HR:0.723, 95% CI: 0.683–0.765, *P* < 0.001) had a significantly decreased risk of death compared to unmarried patients. In stratified analysis, the married patients showed better OS and CCSS than the unmarried patients diagnosed in 1975–2000 and 2001–2017.

**Conclusions:**

Being married was associated with a favorable prognosis of cervical cancer, and marital status was an independent prognostic factor for cervical cancer.

## Background

Cervical cancer is the fourth most common malignant tumor troubling women worldwide [1]. Although the incidence of cervical cancer has been suppressed due to human papillomavirus(HPV)vaccination and cervical cancer screening in many countries and regions [2], there is still a large number of people who die from cervical cancer each year [3]. It was reported that approximately 266,000 deaths were due to cervical cancer per year globally [4, 5]. Moreover, most cervical cancer survivors face several long-term risks, such as recurrence and metastasis [6]. Cervical cancer remains a serious threat to women’s health.

It was reported that the prognosis of cervical cancer was associated with many factors, such as Tumor-Node-Metastasis (TNM) stage, grade, tumor invasion, and lymph node involvement et al. [7–9]. In recent years, more and more attention has been paid to the impact of psychosocial factors on the prognosis of tumor patients [10, 11]. In particular, marital status has been shown to be an essential psychosocial factor affecting long-term outcomes in various tumors, such as breast cancer [12], rectal cancer [13], hepatocellular carcinoma [14], cervical cancer [15], ovarian cancer [16], and several other types of cancers [17, 18]. Although some studies showed that marriage appears to benefit the survival in patients with cervical cancer [19], the relationship between marital status and the prognosis of cervical cancer has not been fully elucidated due to the lack of large sample studies. Therefore, examining the effect of marital status on the prognosis of cervical cancer patients is urgently needed.

However, previous studies mainly focused on traditional survival analyses such as standard Kaplan-Meier and Cox regression methods, and these studies do not consider other cause-specific death as a competing event to cervical cancer-specific death, thus leading to an overestimated risk of cervical cancer-specific death [20, 21]. Therefore, a competing risk regression model was utilized to investigate the efficacy of marital status on the prognosis of cervical cancer patients to reduce this bias.

Here, we conducted a retrospective study using the SEER database. The efficiency of marital status on the long-term survival of cervical cancer patients was analyzed through several statistical methods, such as the Kaplan-Meier, Cox regression, and Competing risk regression models. This study would provide guidance on the prognosis of cervical cancer for clinicians and patients to help the decision-making of follow-up treatment.

## Materials and methods

### Patients section

All data was obtained from the SEER database by SEER*Stat 8.4.0. (https://seer.cancer.gov/seerstat/). The SEER database is a publicly available, federally funded cancer reporting system [22], collecting patients’ information in 18 tumor registries and covering approximately 28% of the total U.S. population [23]. We obtained signed authorization and permission from the SEER program to access and use the data (10,762-Nov2021), and followed the agreement throughout the process to protect the privacy of patients.

**Fig. 1:**
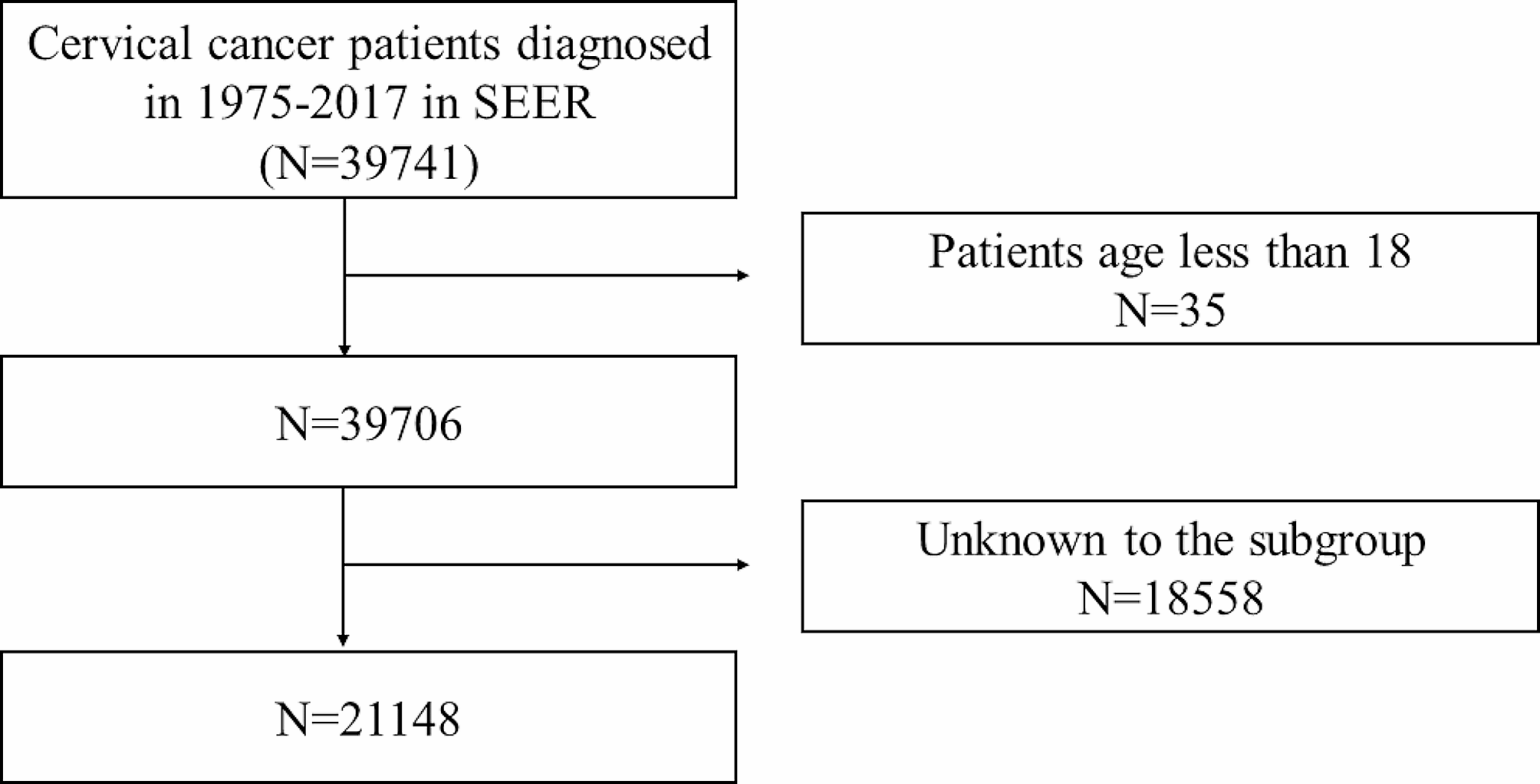
Flow chart of the patient’s enrollment and exclusion. Draw.io software (version 20.3.0, JGraph Ltd.) was used for figure creation

In this study, female patients who had been diagnosed with cervical cancer between 1975 and 2017 were first included, and cervical cancer patients were identified according to the International Classification of Diseases of Oncology, Third edition, (ICD-O-3) codes: C53.0, C53.1, C53.8, and C53.9. Then, a series of screening criteria for the patients initially included were carried out; the details are shown as a flowchart in Fig. 1. Overall, 21,148 patients were enrolled in this study, and all were classified into the married and unmarried groups by marital status. Divorced, widowed, and separated status in marriage were considered unmarried.

Variables included the demographic information (year of diagnosis, age, race, and marital status at diagnosis), pathologic and histologic information (grade, histology, stage, and regional nodes), clinical treatments (surgery, radiotherapy, and chemotherapy), and survival information (survival time and status). The year of diagnosis was divided into the 1975–2000 and 2001–2017 subgroups. Age was divided into < 45, 45–59, and ≥ 60 years subgroups. The race included the White, Black, and other race subgroups.

### Statistical analysis

Excel software was used to organize the data preliminarily, and the chi-square test was used to compare the baseline characteristics of each group. The Propensity score matching (PSM) method was utilized to match the married patient with the unmarried patient by 1:1 using the following characteristics: year of diagnosis, age, race, histology, grade, stage, regional nodes, surgery, radiotherapy, and chemotherapy status. Overall survival (OS) and cervical cancer-specific survival (CCSS) were analyzed using the Kaplan-Meier method, and the differences between survival curves were analyzed by the log-rank test. Cox proportional hazards regression models were used to obtain hazard ratios (HR) and their corresponding 95% confidence intervals (CI) for prognostic factors for OS and CCSS. In the multivariate Cox regression analyses, a stepwise procedure was employed to retain the most significant prognostic factors. To further control the confounding factors, the stratified analysis according to the year of diagnosis was conducted using the Kaplan-Meier method.

Deaths were classified into cervical cancer-specific death (CCSD) and other causes-specific death (OCSD) by the cause of death. The Fine and Gray competing risk model was used to reduce the estimation bias by dividing causes of death into two subgroups. The cumulative incidence function (CIF) and Gray’s test were performed to identify and assess statistical probability differences resulting from competing risk events. The PSM method, the chi-square test, the Kaplan-Meier survival analysis, and the Cox regression analysis were performed using SPSS-IBM 26.0 software (Chicago, IL, USA). The competing risk regression analysis was conducted in the R software (version 4.0.2) using the R package cmprsk. *P* < 0.05 was considered statistically significant.

## Results

### Patient demographics and clinical characteristics

A total of 21,148 out of 39,741 patients with cervical cancer were included in the current study. Among these patients, there were 10,603 married patients and 10,545 unmarried patients. The patients’ baseline characteristics are summarized in Table 1. 11,720 (55.4%) patients were diagnosed between 1975 and 2000, and 9428 (44.6%) patients were diagnosed between 2001 and 2017. The number of patients < 45, 45–59, ≥ 60 years of age were 9000 (42.6%), 6051 (28.6%) and 6097(28.8%); 16,092 (76.1%) patients were white race, 2241 (10.6%) patients were black race and 2815 (13.3%) patients were other races. There were 14,314 (67.6%) squamous cell carcinoma, 4024 (19.9%) adenocarcinoma, and 2630 (12.4%) other histologic types. The numbers of patients in histological grades with well, moderate and poor differentiation/undifferentiation were 3044 (14.4%), 8739 (41.3%) and 9365(44.3%), respectively. The numbers of patients in stages with localized, regional, and distant tumors were 10,432 (49.3%), 8385(41.3%), and 9365 (44.3%), respectively. 1716 (8.1%) patients were positive regional nodes, and 19,432 (91.9%) were negative regional nodes. A total of 13,076(61. 8%) patients received surgery, 12,531 (59.3%) patients received radiotherapy, and 6117 (28.9%) patients received chemotherapy. By comparing patients in the married and unmarried groups, significant differences (*p* < 0.05) were found in the year of diagnosis, age, race, grade, stage, histologic type, regional nodes, surgery, radiotherapy, and chemotherapy.

**Table 1:** Baseline demographic and tumor characteristics of married and unmarried patients

	Before PSM				After PSM			
Characteristics	Total *n* = 21,148(%)	married *n* = 10,603(%)	unmarried *n* = 10,545(%)	*P* value	Total *n* = 14,412(%)	married *n* = 7206(%)	unmarried *n* = 7206(%)	*P* value
Year of diagnosis				< 0.001				1.00
1975–2000	11,720(55.4%)	6101(57.5%)	5619(53.3%)		7780(54.0%)	3890(54.0%)	3890(54.0%)	
2001–2017	9428(44.6%)	4502(42.5%)	4926(46.7%)		6632(46.0%)	3316(46.0%)	3316(46.0%)	
age				< 0.001				1.00
< 45	9000(42.6%)	5035(47.5%)	3965(37.6%)		6502(45.1%)	3251(45.1%)	3251(45.1%)	
45–59	6051(28.6%)	3349(31.6%)	2702(25.6%)		4144(28.8%)	2072(28.8%)	2072(28.8%)	
≥ 60	6097(28.8%)	2219(20.9%)	3878(36.8%)		3766(26.1%)	1883(26.1%)	1883(26.1%)	
Race				< 0.001				1.00
White	16,092(76.1%)	8345(78.7%)	7747(73.5%)		11,678(81.0%)	5839(81.0%)	5839(81.0%)	
Black	2241(10.6%)	646(6.1%)	1595(15.1%)		1046(7.3%)	523(7.3%)	523(7.3%)	
Others	2815(13.3%)	1612(15.2%)	1203(11.4%)		1688(11.7%)	844(11.7%)	844(11.7%)	
Histologic type				< 0.001				1.00
SCC	14,314(67.6%)	6776(63.9%)	7538(71.5%)		10,374(72.0%)	5187(72.0%)	5187(72.0%)	
AC	4204(19.9%)	2460(23.2%)	1744(16.65%)		2522(17.5%)	1261(17.5%)	1261(17.5%)	
Other	2630(12.4%)	1367(12.9%)	1263(12.0%)		1516(10.5%)	758(10.5%)	758(10.5%)	
Grade				< 0.001				1.00
Well	3044(14.4%)	1739(16.4%)	1305(12.4%)		1900(13.2%)	950(13.2%)	950(13.2%)	
Moderately	8739(41.3%)	4360(41.1%)	4379(41.5%)		6238(43.3%)	3119(43.3%)	3119(43.3%)	
Poorly/Undiff	9365(44.3%)	4505(42.5%)	4861(46.1%)		6274(43.5%)	3137(43.5%)	3137(43.5%)	
Stage				< 0.001				1.00
Localized	10,432(49.3%)	5746(54.2%)	4686(44.54%)		7548(52.4%)	3774(52.4%)	3774(52.4%)	
Regional	8385(39.6%)	3834(36.2%)	4551(43.2%)		5624(39.0%)	2812(39.0%)	2812(39.0%)	
Distant	2331(11.0%)	1023(9.6%)	1308(12.4%)		1240(8.6%)	620(8.6%)	620(8.6%)	
Regional nodes				0.004				1.00
Positive	1716(8.1%)	918(8.7%)	798(7.6%)		1036(7.2%)	518(7.2%)	518(7.2%)	
Negative	19,432(91.9%)	9685(91.3%)	9747(92.4%)		13,376(92.8%)	6688(92.8%)	6688(92.8%)	
Surgery				< 0.001				1.00
Yes	13,076(61.8%)	7233(68.2%)	5843(55.4%)		9134(63.4%)	4567(63.4%)	4567(63.4%)	
No	8072(38.2%)	3370(31.8%)	4702(44.6%)		5278(36.6%)	2639(36.6%)	2639(36.6%)	
Radiotherapy				< 0.001				
Yes	12,531(59.3%)	5906(55.7%)	6625(62.8%)		8500(59.0%)	4250(59.0%)	4250(59.0%)	1.00
No	8617(40.7%)	4697(44.3%)	3920(37.2%)		5912(41.0%)	2956(41.0%)	2956(41.0%)	
Chemotherapy				< 0.001				1.00
Yes	6117(28.9%)	2886(27.2%)	3231(30.6%)		4122(28.6%)	2061(28.6%)	2061(28.6%)	
No	15,031(71.1%)	7717(72.8%)	7314(69.4%)		10,290(71.4%)	5145(71.4%)	5145(71.4%)	

PSM, Propensity score matching; SCC, Squamous Cell Carcinoma; AC, Adenocarcinoma

After the PSM, a total of 14,412 patients were included, of which 7206 were married and 7206 were unmarried, and no significant differences were found in the covariates mentioned above (Table 1).

### Marital status and survival analysis

**Fig. 2:**
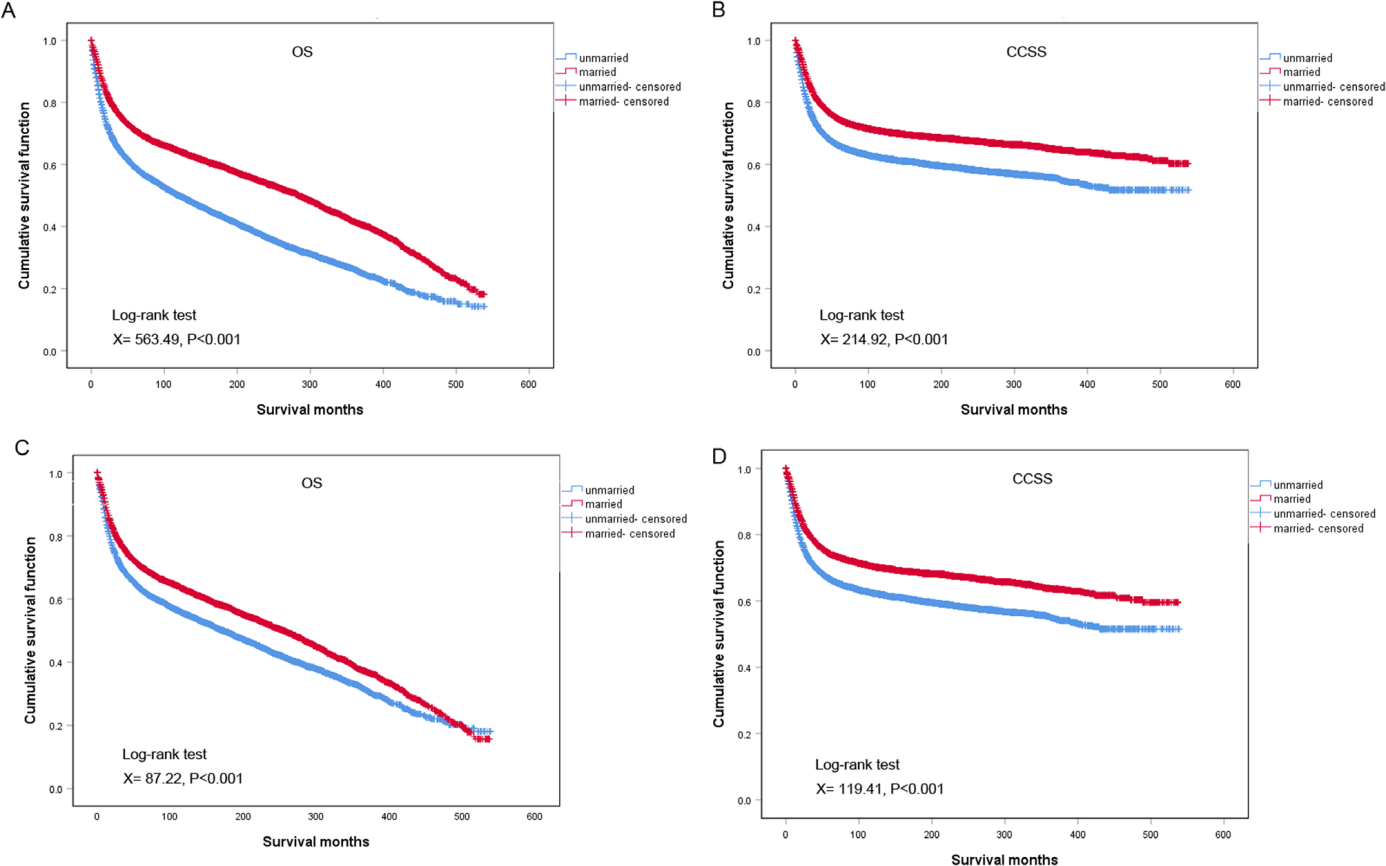
OS and CCSS survival curves of patients with cervical cancer in different marital statuses. **(AB)** Before PSM; **(CD)** After PSM. OS: overall survival; CCSS: cervical cancer-specific survival; PSM: propensity score matching

Kaplan–Meier curves showed significant differences in OS (*P* < 0.001, Fig. 2A) and CCSS (*P* < 0.001, Fig. 2B) outcomes between the married and unmarried patients. Married patients had better survival outcomes than unmarried patients. The crude median survival among married patients was higher (284 months, range 0- 538) than among unmarried patients (120 months, range 0- 539). Moreover, the 5-year OS and CCSS for married patients were 71.0% and 74.4%, while 59.0% and 66.6% for unmarried patients. After PSM, the 5-year OS and CCSS for married patients were 70.2% and 74.2%, while 63.1% and 66.6% for unmarried patients, and married patients still had a significant survival advantage compared to unmarried patients (Fig. 2C and D). These results indicated that marriage could confer OS and CCSS benefits for patients with cervical cancer.

### Cox regression model analysis

To further investigate whether marital status is an independent prognostic factor in OS and CCSS, the univariate and multivariate Cox regression analysis were conducted. Married patients had significantly higher odds of survival (OS HR: 0.830, 95% CI: 0.798–0.862; CCSS HR: 0.892, 95% CI: 0.850–0.937) compared to the unmarried patient, which suggested that marital status was an independent prognostic factor for both OS and CCSS. In addition, several covariates, including year of diagnosis, age, race, histologic type, grade, stage, and surgery, were also the independent prognostic factors of OS and CCSS (Table 2).

**Table 2:** Univariate and multivariate Cox regression model analysis of OS and CCSS

	OS				CCSS			
Characteristics	Univariate analysis		Multivariate analysis		Univariate analysis		Multivariate analysis	
	HR (95% CI)	*P*	HR (95% CI)	*P*	HR(95% CI)	*P*	HR(95% CI)	*P*
Marital status								
Unmarried	1		1		1		1	
Married	0.638(0.614,0.662)	0.000	0.830(0.798, 0.862)	0.000	0.706(0.673, 0.740)	0.000	0.892(0.850, 0.937)	0.000
Year								
1975–2000	1		1		1		1	
2001–2017	0.820(0.786, 0.855)	0.000	0.826 (0.786, 0.869)	0.000	0.849(0.809, 0.892)	0.000	0.797(0.751, 0.846)	0.000
age								
< 45	1		1		1		1	
45–59	2.183(2.076, 2.295)	0.000	1.606 (1.525, 1.691)	0.000	1.683(1.585, 1.786)	0.000	1.089(1.024, 1.158)	0.007
≥ 60	4.751(4.529, 4.983)	0.000	2.896 (2.751, 3.049)	0.000	2.660(2.512, 2.816)	0.000	1.377(1.295, 1.464)	0.000
Race								
White	1		1		1		1	
Black	1.407(1.331, 1.489)	0.000	1.187 (1.122, 1.257)	0.000	1.394(1.300, 1.495)	0.000	1.192(1.110, 1.280)	0.000
Other	0.879(0.828, 0.932)	0.000	0.825 (0.778, 0.876)	0.000	0.929(0.864, 0.998)	0.044	0.891(0.829, 0.958)	0.002
Histologic type								
SSC	1		1		1		1	
AC	0.738(0.702, 0.777)	0.000	1.137 (1.078, 1.199)	0.000	0.782(0.734, 0.833)	0.000	1.304(1.220, 1.394)	0.000
Others	1.036(0.978, 1.097)	0.231	1.210 (1.141, 1.283)	0.000	1.231(1.151, 1.317)	0.000	1.398(1.304, 1.498)	0.000
Grade								
Well	1		1		1		1	
Moderately	1.555(1.454, 1.662)	0.000	1.153 (1.076, 1.235)	0.000	1.960(1.781, 2.157)	0.000	1.343(1.218, 1.482)	0.000
Poorly/Undiff	2.318(2.172, 2.473)	0.000	1.386 (1.295, 1.484)	0.000	3.257(2.968, 3.574)	0.000	1.716(1.558, 1.890)	0.000
Stage								
Localized	1		1		1		1	
Regional	3.047(2.920, 3.180)	0.000	1.892 (1.797, 1.991)	0.000	4.517(4.248, 4.803)	0.000	3.062(2.845, 3.295)	0.000
Distant	9.461(8.942, 10.009)	0.000	5.715 (5.351, 6.103)	0.000	16.411(15.291, 17.613)	0.000	10.472(9.627, 11.392)	0.000
Regional nodes								
Negative	1		1		1		1	
Positive	1.155(1.079, 1.237)	0.000	1.037 (0.961, 1.119)	0.354	1.391(1.289, 1.501)	0.000	1.027(0.942, 1.120)	0.547
Surgery								
No	1		1		1			
Yes	0.266(0.256, 0.276)	0.000	0.496 (0.472, 0.521)	0.000	0.236(0.225, 0.248)	0.000	0.429(0.403, 0.456)	0.000
Radiotherapy								
No	1		1		1		1	
Yes	2.882(2.760, 3.010)	0.000	1.065 (1.009, 1.125)	0.023	3.122(2.948, 3.305)	0.000	0.964(0.899, 1.034)	0.310
Chemotherapy								
No	1		1		1		1	
Yes	1.651(1.584, 1.721)	0.000	0.950(0.900, 1.003)	0.062	1.985(1.891, 2.083)	0.000	0.948(0.889, 1.010)	0.101

### The competing risk model analysis of CCSD and OCSD

**Fig. 3:**
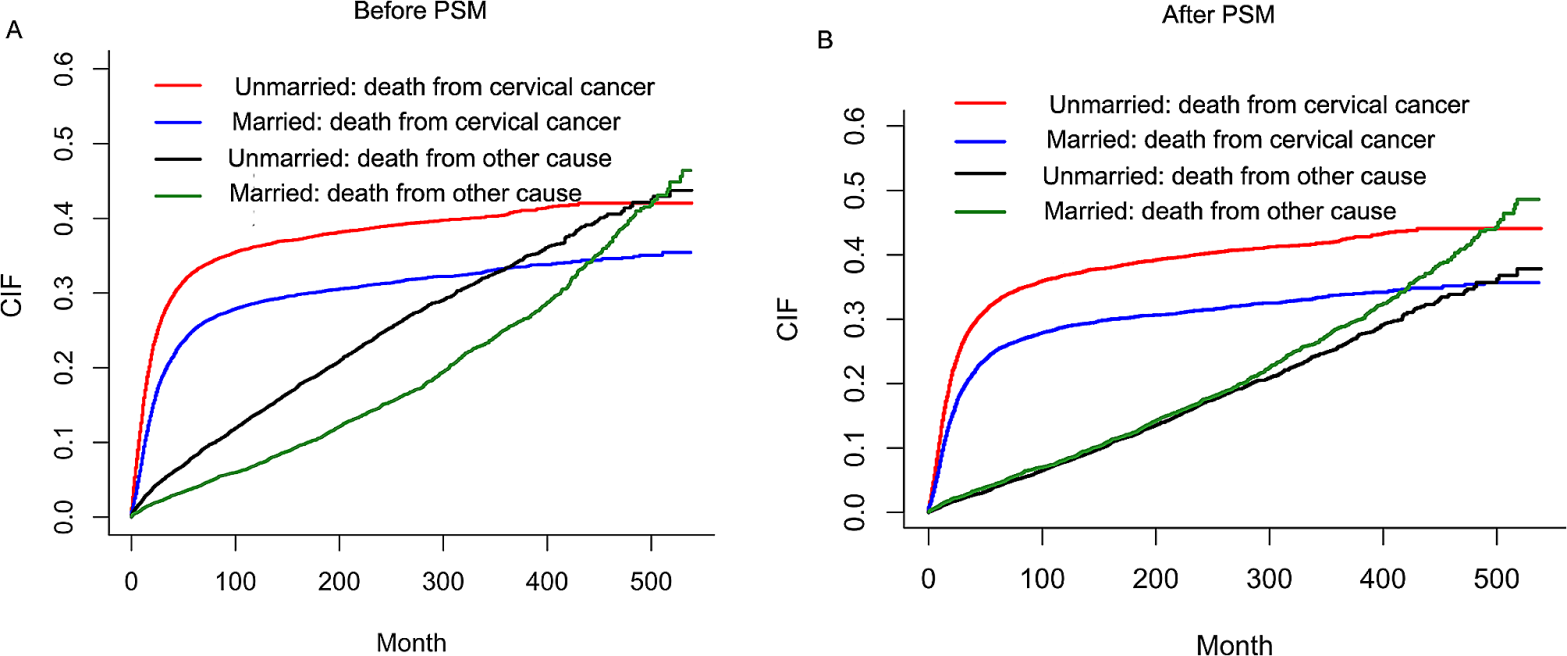
Cumulative incidence estimates of CCSD and OCSD of married and unmarried patients. **(A)** Cumulative incidence curve of CCSD and OCSD for patients before PSM; **(B)** Cumulative incidence curve of CCSD and OCSD for patients after PSM. CCSD: cervical cancer-specific death; OCSD: other cause-specific death; PSM: propensity score matching

Before PSM, the total cumulative incidence of cervical cancer-specific death (CCSD) was 29.93% (3173/10,603) in the married group and 36.83% (3884/10,545) in the unmarried group. While the total cumulative incidence of other cause-specific death (OCSD) was 16.67% (1768/10,603) for patients in the married group and 21.76% (2295/10,545) for those in the unmarried group. The cumulative CCSD and OCSD rates at five years are 25.6% and 3.40% for patients in the married group, respectively, while 34.0% and 7.0% for those in the unmarried group, respectively. The married patients had better cumulative CCSD incidence (HR:0.743, 95% CI:0.708–0.778, *P* < 0.001) and OCSD incidence (HR: 0.678, 95% CI:0.638–0.721, *P* < 0.001) than the unmarried patients (Fig. 3A).

After PSM, the total cumulative incidence of CCSD was 29.92% (2156/7206) in the married group and 38.05% (2742/7206) in the unmarried group, while the total cumulative incidence of OCSD was 17.97% (1295/7206) for patients in the married group, and 15.93% (1148/7206) for those in the unmarried group. The cumulative CCSD and OCSD rates at five years were 25.8% and 4.0% for patients in the married group, respectively, while 33.4% and 3.5% for those in the unmarried group, respectively. As shown in Fig. 3B, the married group had better cumulative CCSD incidence (HR:0.723, 95% CI: 0.683–0.765, *P* < 0.001) and worse OCSD incidence (HR: 1.137, 95% CI: 1.052–1.230, *P* = 0.001).

Furthermore, multivariate competing risk regression model analysis showed that the married patients had significantly decreased risk of CCSD (HR: 0.831, 95% CI: 0.781–0.885, *P* < 0.001) and OCSD (HR: 0.927, 95% CI: 0.880–0.976, *P* = 0.004) compared to the unmarried patients (Table 3), which suggested that marriage was a better prognostic indicator of cervical cancer. In addition, several covariates, including year of diagnosis, age, race, histologic type, grade, stage, and radiotherapy, were also significantly associated with CCSD and OCSD.

**Table 3:** Multivariate competing risk model analysis of CCSD and OCSD.

Characteristics	CCSD		OCSD	
	HR (95% CI)	*P*	HR (95% CI)	*P*
Marital status				
Unmarried	1		1	
Married	0.927(0.880, 0.976)	0.004	0.831(0.781, 0.885)	0.000
Year of diagnosis				
1975–2000	1		1	
2001–2017	0.751(0.704, 0.801)	0.000	0.615 (0.563, 0.671)	0.000
age				
< 45	1		1	
45–59	1.032 (0.968, 1.100)	0.332	2.864 (2.615, 3.137)	0.000
≥ 60	1.111 (1.041, 1.186)	0.002	6.432 (5.886, 7.029)	0.000
Race				
White	1		1	
Black	1.164 (1.078, 1.260)	0.000	1.138 (1.035, 1.252)	0.008
Other	0.917 (0.851, 0.988)	0.023	0.788 (0.713, 0.871)	0.000
Histologic type				
SSC	1		1	
AC	1.291 (1.203, 1.385)	0.000	0.756 (0.694, 0.824)	0.000
Other	1.393 (1.288, 1.505)	0.000	0.721 (0.645, 0.806)	0.000
Grade				
Well	1		1	
Moderately	1.338 (1.213, 1.476)	0.000	0.869 (0.791, 0.954)	0.003
Poorly/Undiff	1.673 (1.517, 1.845)	0.000	0.859 (0.781, 0.945)	0.002
Stage				
Localized	1			
Regional	2.743 (2.536, 2.967)	0.000	0.732 (0.677, 0.791)	0.000
Distant	8.639 (7.865, 9.489)	0.000	0.258(0.218, 0.306)	0.000
Regional nodes				
Negative				
Positive	0.998 (0.901, 1.084)	0.799	0.901(0.761, 1.068)	0.229
Surgery				
No				
Yes	1.162 (1.070, 1.262)	0.000	1.065 (0.984, 1.152)	0.117
Radiotherapy				
No				
Yes	1.046 (0.973, 1.124)	0.000	1.309 (1.210, 1.416)	0.000
Chemotherapy				
No				
Yes	0.927 (0.880, 0.976)	0.221	0.751 (0.677, 0.833)	0.000

### Survival analysis of marital status in year of diagnosis subgroups

**Fig. 4:**
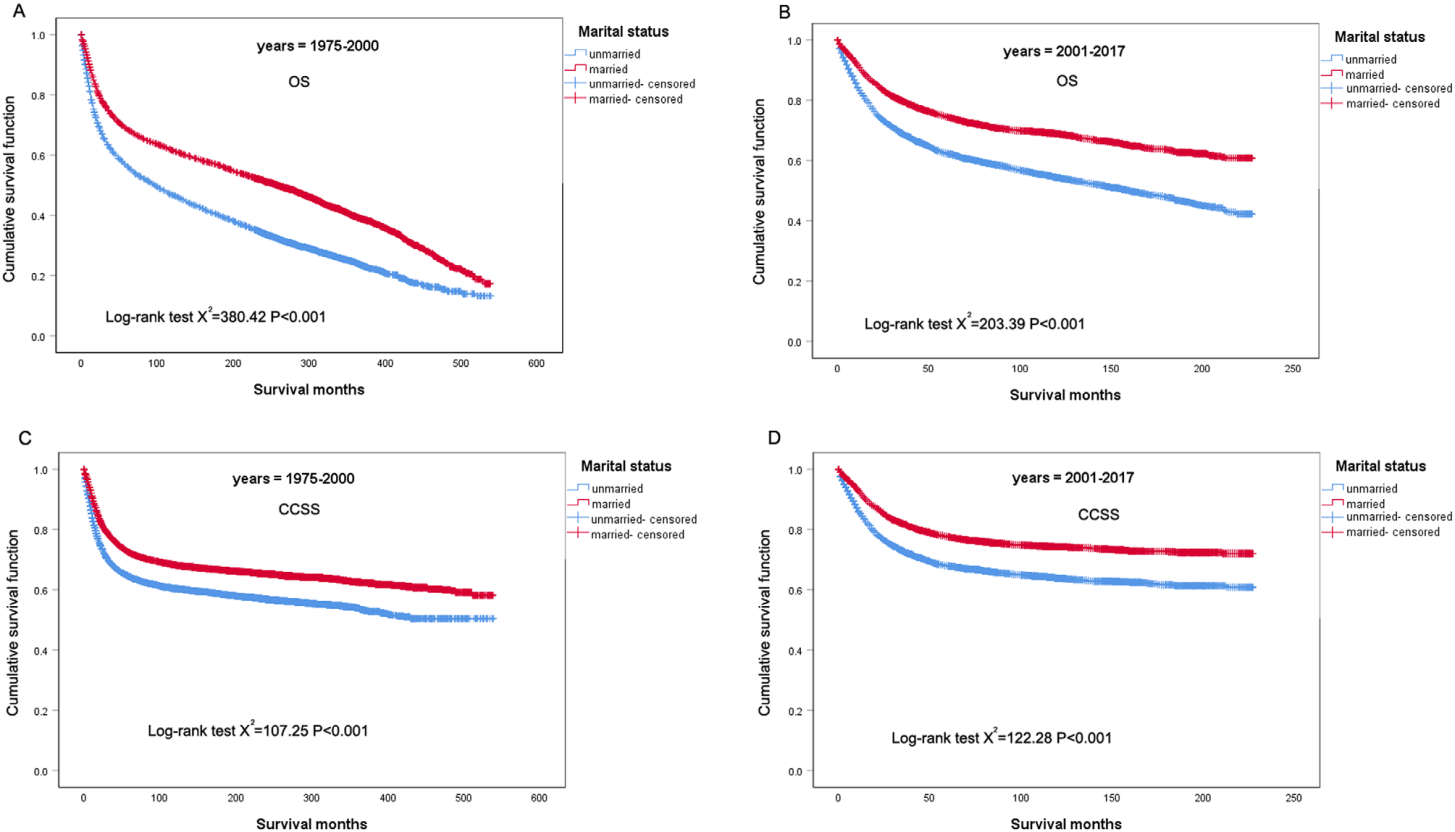
OS and CCSS survival curves of patients with cervical cancer in the year of diagnosis subgroups. **(A)** OS survival curves in 1975–2000 subgroup; **(B)** OS survival curves in 2001–2017 subgroup; **(C)** CCSS survival curves in 1975–2000 subgroup; **(D)** CCSS survival curves in 2001–2017 subgroup; OS: overall survival; CCSS: cervical cancer-specific survival; PSM: propensity score matching

**Fig. 5:**
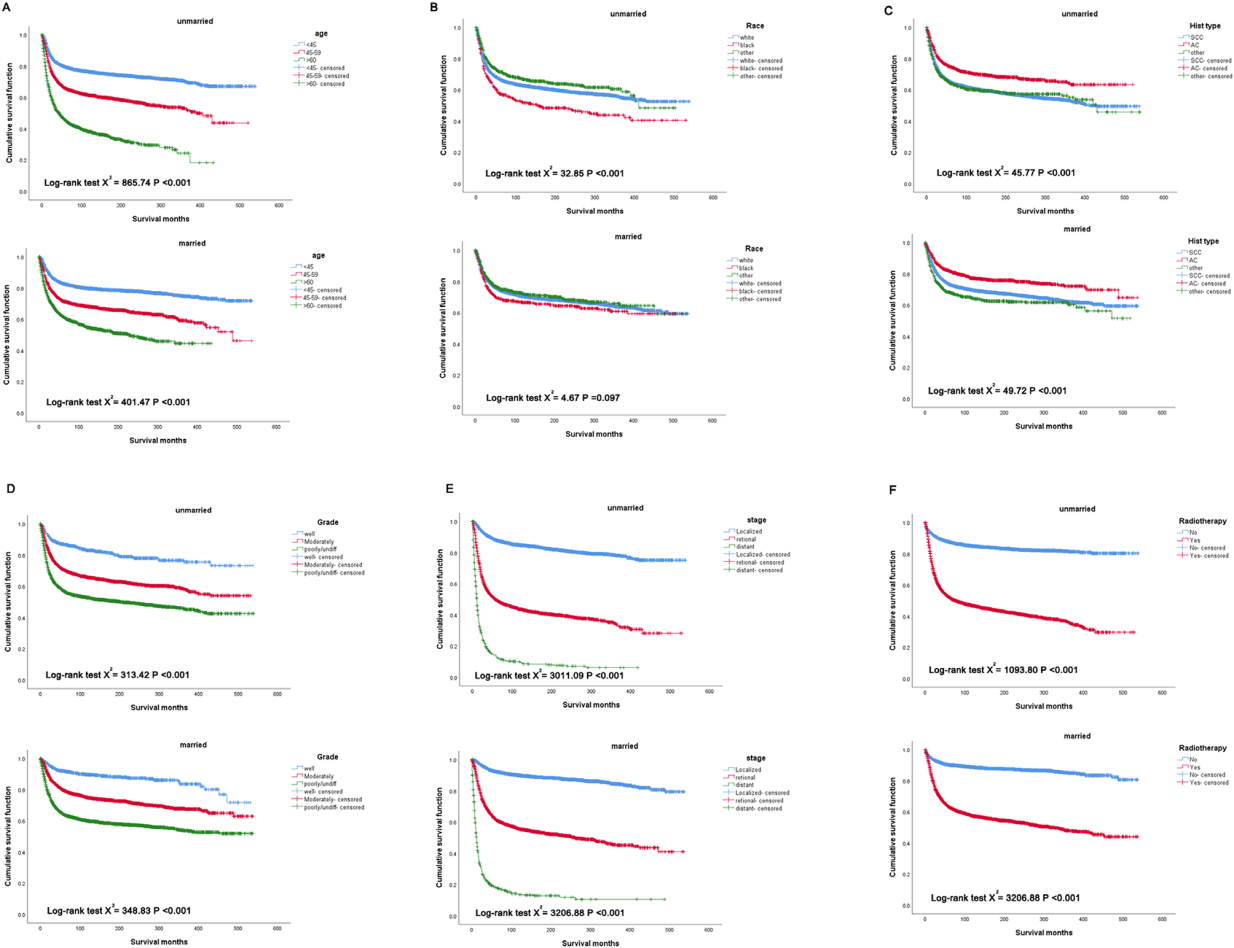
Kaplan-Meier subgroup analysis of cervical cancer patients according to marital status. **(A)** Survival curves of cervical cancer patients with different ages under different marital status. **(B)** Survival curves of cervical cancer patients with different races under different marital status. **(C)** Survival curves of cervical cancer patients with different histologic types under different marital status. **(D)** Survival curves of cervical cancer patients with different grade under different marital status. **(E)** Survival curves of cervical cancer patients with different stage under different marital status. **(F)** Survival curves of cervical cancer patients with radiotherapy under different marital status

To further investigate whether the effect of marital status on the prognosis of cervical cancer is related to the period, patients were divided into two subgroups (1975–2000 and 2001–2017) according to the year of diagnosis, and then stratified analysis was performed using the Kaplan-Meier method. As shown in Fig. 4A and B, the married patients all showed significantly better OS prognosis than the unmarred patients diagnosed in 1975–2000(HR: 0.642, 95% CI: 0.613–0.671, *P* < 0.001) and 2001–2017(HR: 0.612, 95% CI: 0.571–0.655, *P* < 0.001). Similar to the above results, the married patients all had better CCSS than the unmarried patients diagnosed in 1975–2000(HR: 0.733, 95% CI: 0.691–0.778, *P* < 0.001), (Fig. 4C) and 2001–2017(HR: 0.648, 95% CI: 0.600-0.701, *P* < 0.001), (Fig. 4D). Additionally, subgroup analysis according to marital status showed that age, histologic type, grade, stage, and radiotherapy were significantly correlated with CCSS regardless of marital status, however, the association between race and CCSS was different in the married and unmarried subgroup (Fig. 5), which further confirmed the effect of marital status on the prognosis of cervical cancer.

## Discussion

In this study, we investigated the association between marital status and the long-term survival prognosis of cervical cancer patients by analyzing a cohort of 21,148 cervical cancer patients in the SEER database from 1975 to 2017. We demonstrated that being married was significantly associated with a better prognosis for patients with cervical cancer.

Recently, marital status, as a social and psychological factor, has attracted increasing attention in the prognosis of cancer [24]. It was reported that marital status has emerged as a significant influence on several cancer outcomes, such as medullary thyroid cancer [17], Liver cancer [25], and breast cancer [26] et al. In the current study, 50.14% (10,603/21,148) patients were married, and 49.86% (10,545/21,148) were unmarried. Consistent with previous studies, we found that married patients had better OS and CCSS than unmarried patients by Kaplan-Meier analysis. The HR of OS (HR: 0.830, 95%CI: 0.798–0.862) and CCSS (HR: 0.892, 95% CI: 0.850–0.937) by Cox regression analysis implied that marriage was a good prognostic factor of cervical cancer. Moreover, compared to these previous studies [15], we included more patients and extended periods. Some clinicopathological characteristics, such as age, grade, tumor stage, and histologic type, have been identified as important factors affecting the prognosis of patients with cervical cancer [27, 28]. Some research showed that the tumorigenesis of cervical cancer is very complex and involves different human papillomavirus genotypes, molecular pathways, DNA hypermethylation patterns, and oncogenes expression [29, 30]. In this study, we found that age, race, histologic type, grade, and stage were independent prognostic factors of cervical cancer by multivariate Cox regression analysis of OS and CCSS. Moreover, the survival advantage of married patients over unmarried patients was further confirmed after controlling the confounding factors by PSM. Therefore, these results indicated that marriage tended to prolong the long-term survival of patients with cervical cancer. Marriage is associated with improved socioeconomic status, especially for women, and married women are more likely to benefit from financial and social support, which is positively related to the prognosis of cancer [31, 32].

Nevertheless, the competing risk, which could disturb cancer-specific death [33] and hamper the emergence of the primary event attributed to the estimation bias arising from OCSD, should not be neglected. To eliminate the potential estimation bias of CCSD, which had been considered one of the most valuable prognostic indicators for cervical cancer [6], univariate and multivariate competing risk regression models were carried out. We found that the cumulative incidence of CCSD in the married group was significantly lower than the unmarried group before and after PSM. Univariate competing risk regression analysis showed that the married patients had significantly decreased risk of CCSD before (HR:0.743, 95% CI:0.708–0.778, *P* < 0.001) and after PSM(HR: 0.831, 95% CI: 0.781–0.885, *P* < 0.001) compared to the unmarried patients after accounting for the competing risk of OCSD. Moreover, multivariate competing risk regression analysis further confirmed the significant association of marital status with CCSD (HR: 0.831, 95% CI: 0.781–0.885, *P* < 0.001). Meanwhile, we also noticed that after PSM, the risk of OCSD in the married group was higher than the unmarried group, suggesting that being married may be related to the risk of OCSD. However, the exact cause was unknown, because there are many contributing factors to OCSD. Therefore, further study was worthwhile.

Additionally, a subgroup analysis based on the year of diagnosis was conducted to account for the long period of patient inclusion in this study and differences in medical technology and living conditions across different time periods. A sustained advantage of married patients in terms of survival was still observed compared to unmarried patients. These findings confirmed the Cox regression analysis result and were consistent with previous reports [15, 34]. Some psychosocial and socioeconomic factors may contribute to the association between marital status and the prognosis of cervical cancer, and the existence of marriage means more financial and emotional support to help deal with potential emotional distress and anxiety when coping with cancer and then improves the survival period of patients [15, 35, 36]. However, the underlying mechanisms are not entirely understood. Indeed, many factors affect the prognosis of patients with cervical cancer. A growing body of evidence suggested that local surgical treatment of cervical intraepithelial neoplasia(CIN) reduces the risk of treatment failure but increases the risk of adverse obstetric outcomes, including preterm birth, low birth weight, premature rupture of the membranes et al. [37, 38]. Therefore, the balanced treatment effectiveness and reproductive morbidity also need to be considered for women with family planning. In addition, it was reported that prophylactic HPV vaccination at the time of local surgical treatment for high-grade CIN might reduce the risk of recurrence, but the evidence is insufficient [39, 40]. Large-scale, high-quality randomized controlled trials are required.

## Conclusions

In summary, our study demonstrated that the existence of marriage could reduce the risk of CCSD and improve the OS and CCSS of patients with cervical cancer. Marital status significantly affects the prognosis of cervical cancer. These can help patients, doctors, and researchers better deal with the prognosis of cervical cancer. However, a clear definitive explanation of such an advantage has yet to be determined, and further studies are needed to investigate the possible cause of being married, which is associated with a good prognosis of cervical cancer.

## Data Availability

Data from the SEER program is available for the public. The data supporting the conclusions of this article are available in the SEER database (https://seer.cancer.gov/).

## Abbreviations

AJCC: American Joint Committee on Cancer
CCSS: Cervical cancer specific survival
CCSD: Cervical cancer-specific death
OS: Overall survival
OCSD: Other cause-specific death
PSM: Propensity score matching
HPV: Human papillomavirus
SEER: Surveillance, Epidemiology, and End Results database
HR: Hazard ratio
ICD‑O‑3: International Classification of Diseases for Oncology, third edition morphology code
